# Beyond the pale: Insights into hypopigmented mycosis fungoides - A case report

**DOI:** 10.51866/cr.644

**Published:** 2024-08-17

**Authors:** Zauddin Nur Zafirah, Azwanis Abdul Hadi

**Affiliations:** 1 MBBS, Department of Family Medicine, Faculty of Medicine , International Islamic University Malaysia, Jalan Sultan Ahmad Shah, Kuantan, Pahang, Malaysia. Email: zafirahzauddin@gmail.com; 2 MBChB, M.Med (FamMed), Department of Family Medicine, Faculty of Medicine, International Islamic University Malaysia, Jalan Sultan Ahmad Shah, Kuantan, Pahang, Malaysia.

**Keywords:** Mycosis fungoides, Hypopigmentation, Skin neoplasm

## Abstract

Hypopigmented mycosis fungoides (MF) is a rare variant of cutaneous T-cell lymphoma, a type of extranodal non-Hodgkin lymphoma. This report presents the case of a 9-year-old boy with a 2-year history of asymptomatic, hypopigmented skin lesions that were resistant to topical treatment. He was initially treated for a fungal skin infection and had received multiple courses of topical antifungals and steroids but showed no improvement, which led to further evaluation and a referral to a dermatologist. A skin biopsy was performed, and the diagnosis of hypopigmented MF was confirmed through skin histopathology and immunohistochemistry study. His lesions responded well to cycles of narrowband ultraviolet B phototherapy, showing almost complete clearance after 4 months without any side effects.

## Introduction

Hypopigmented skin lesions are a common complaint in paediatric-age groups presenting to outpatient clinics. These age groups are brought by their parents to seek medical attention due to clinically apparent concerns and cosmetic issues. The common clinical diagnoses for such hypopigmented lesions are pityriasis alba, tinea versicolor, post- inflammatory hypopigmentation and vitiligo. In endemic areas, tuberculoid leprosy should be considered in the differential diagnosis. In addition to these conditions, another rare but important diagnosis to be considered is hypopigmented mycosis fungoides (MF), which is a variant of MF. MF is the most common type of primary cutaneous T-cell lymphoma (CTCL), accounting for about 62% of CTCL cases, and belongs to the group of extranodal non-Hodgkin lymphomas.^1^ Herein, we present a case wherein a patient with seemingly common hypopigmented lesions was found to have skin malignancy.

## Case presentation

A 9-year-old boy presented with a complaint of progressive, multiple hypopigmented lesions that developed over his body for the past 2 years. Initially, they appeared as small, red lesions on his back and spread to his anterior trunk. Over time, the lesions faded into whitish patches and expanded to include the back and front of the trunk, the inguinal region and the face, sparing the upper and lower limbs. There was neither itching nor pain on the affected areas. The patient had not experienced any prolonged fever, lymph node swelling or bleeding tendency. There was no family history of atopy or malignancy.

His mother brought him to local health clinics, where he was given courses of topical antifungal medication and local steroids, but which yielded no improvement. He was then referred to a dermatology clinic for further investigation.

**Figure 1:**
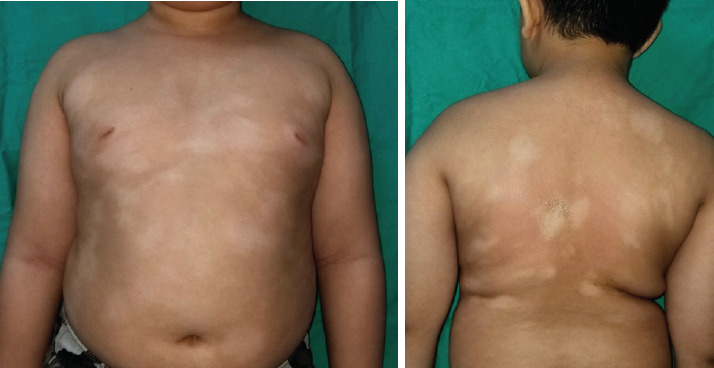

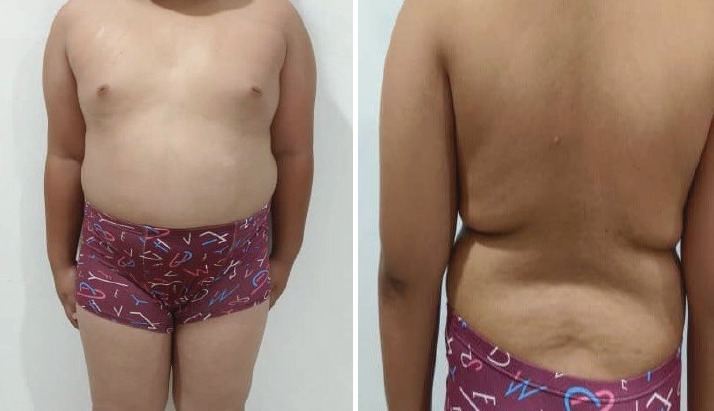
Clinical photographs demonstrating the response of hypopigmented mycosis fungoides lesions to phototherapy. (A) Pre-treatment presentation showing hypopigmented skin patches over the anterior trunk, back and buttocks. (B) Appearance after completed phototherapy, demonstrating significant repigmentation.

On examination, the patient was not dysmorphic. Skin examination revealed multiple hypopigmented macules and patches over the back, abdomen and inguinal area (Figure 1). The lesions were flat, non-itchy and non-scaly with well-demarcated borders. Skin sensation was intact. There was no lymphadenopathy or hepatosplenomegaly. Wood’s lamp examination revealed enhancement of the hypopigmented lesions, with no greenish fluorescent uptake seen.

The initial working diagnoses were pityriasis versicolor, pityriasis alba, leprosy, post- inflammatory hypopigmentation and progressive macular hypomelanosis. His complete blood count test results, liver fUnction and renal profile were normal. Infectious viral screening and syphilis screening showed negative findings.

A skin punch biopsy was then performed on the patient’s back. Histopathological examination revealed superficial perivascular infiltration by atypical lymphoid cells. These cells exhibited epidermotropism, with basilar tagging observed in the focal area. No Pautrier’s microabscesses were present. The cells were small to medium in size, with irregular hyperchromatic nuclei, inconspicuous nucleoli and scant cytoplasm. In addition, a mild admixture of lymphoplasmacytic cells was noted. The epidermis showed mild irregular acanthosis with a basket-woven corneal layer. The skin adnexa appeared unremarkable. Immunohistochemistry studies demonstrated that the atypical lymphoid cells were positive for CD3, CD5 and CD8, with a greater expression of CD8 than CD4. Notably, there was an aberrant loss of CD2 and CD7, while CD20 staining was absent. Considering the histomorphology, immunohistochemical profile and clinical history, the diagnosis was consistent with hypopigmented MF.

The patient was treated with a topical corticosteroid cream applied twice daily and underwent 30 sessions of narrowband ultraviolet B phototherapy. His lesions responded substantially well, showing repigmentation after 10 cycles of phototherapy. Upon completion of the fUll series of phototherapy sessions, only some faint, residual hypopigmented macules were left scattered on the anterior and posterior aspects of his trunk. Two years later, he continued routine follow-up and remained under surveillance post-phototherapy, with no active or new skin lesions observed to date.

## Discussion

MF is a type of non-Hodgkin lymphoma arising from mature T cells that initially manifests on the skin but can potentially spread to the lymph nodes, blood and other organs. There are several distinct clinical subtypes of MF, which include the pustular, granulomatous, purpuric, hyperkeratotic and hypopigmented subtypes. Hypopigmented MF is a rare variant of MF that is characterised by hypopigmented skin lesions and typically presents in younger age groups compared to the classical form.^2^ According to a substantial cohort study, hypopigmented MF constitutes about 3% of all adult cases of MF.^3^ The classical form of MF usually manifests in individuals in their late 50s, while hypopigmented MF predominantly occurs in children or adolescents and may constitute around 17%–59% of all MF cases identified in the paediatric demographic.^4,5^ It is more prevalent in Asian populations and has a predilection towards darker skin phototypes. In a Southeast Asian cohort, comprising 239 patients with MF, hypopigmented MF was identified as the most common subtype, accounting for 33% of cases.^6^

Hypopigmented MF typically appears as circular or irregularly shaped hypopigmented patches or thin plaques with a fine scale. The lesions tend to be asymptomatic or may cause minimal itching and are primarily found on the torso, buttocks and extremities. The diagnosis of hypopigmented MF can pose challenges or be delayed due to its slow progression and similarity to various benign conditions that present with hypopigmented lesions, including vitiligo, tinea corporis, pityriasis versicolor, pityriasis alba, post-inflammatory hypopigmentation, progressive macular hypomelanosis and leprosy.

Pityriasis alba is a common, self-limiting skin condition primarily affecting children and adolescents, particularly those with an atopic history. It typically presents as multiple, asymptomatic, hypopigmented macules and patches predominantly found on the face, upper trunk and upper limbs. Sun exposure can accentuate the appearance of these lesions. Pityriasis alba typically resolves spontaneously, with normal skin pigmentation gradually returning over a few months to several years and with most cases resolving within 1 year. However, if the lesions persist, become symptomatic or exhibit atypical characteristics (e.g. changes in morphology or pigmentation), further evaluation is warranted to rule out other conditions, particularly hypopigmented MF.

In contrast to the hypopigmentation seen in pityriasis alba, pityriasis versicolor presents with lesions that can be either hypopigmented or hyperpigmented. This common, recurrent, superficial fungal infection manifests as multiple macules, patches or thin plaques, most commonly on the upper trunk and proximal upper extremities. A strong clinical suspicion can often be formed based on physical examination alone, and Wood’s lamp examination may reveal yellow to yellow- green fluorescence. However, a definitive diagnosis of pityriasis versicolor requires a potassium hydroxide preparation, which will microscopically reveal the characteristic hyphal and yeast forms of *Malassezia furfur.*

Immunohistochemical analysis is important for diagnosing hypopigmented MF because neoplastic cells in this condition often express CD8, a hallmark feature of hypopigmented MF.^7^ In contrast to classical MF, which is characterised by neoplastic CD4+ T cells, hypopigmented MF typically shows a predominance of CD8+ cells. A few literature sources suggest that the hypopigmentation observed in hypopigmented MF might be a manifestation of the body’s active immune response to the neoplastic or reactive immune cells. Reactive CD8+ cytotoxic T lymphocytes have been implicated in altering and modifying melanocyte functions.^8^ In some cases, histological findings may be inconclusive for hypopigmented MF; therefore, further follow- up and repeated biopsies may be necessary to establish the diagnosis.

Hypopigmented MF carries a better prognosis than conventional MF and responds well to phototherapy.^9^ It is a cutaneous lymphoma that shows a good response to skin-directed therapies, as demonstrated in this case report, and has a favourable prognosis despite a high recurrence rate.^3^ The first-line treatment for hypopigmented MF is photochemotherapy, and there has been success using narrowband ultraviolet radiation in combination with topical corticosteroids. In most cases, repigmentation occurs following treatment.^10^ Despite a high likelihood of recurrence, most patients respond quite favourably to subsequent treatments.^11^ However, some literature reports cases where the disease progressed to the tumour stage and resulted in death despite adequate treatment. Therefore, long-term follow-up is essential, and its potential lethality should not be underestimated.

## Conclusion

Hypopigmented skin lesions are common in children. In a warm and humid country such as Malaysia, fungal infections frequently occur and commonly present as hypopigmented skin lesions; however, not all discolourations of this sort are attributable to fungal causes. Some cases may indeed be more serious and turn out to be skin malignancies, as highlighted in this case report. Although hypopigmented MF usually follows a benign clinical course, it should always be treated as a malignant neoplastic skin disease. This underscores the importance of a comprehensive clinical examination along with a thorough investigation to ensure accurate diagnosis and appropriate treatment. If a patient presents with a hypopigmented skin lesion that is resistant to standard courses of treatment, it should raise concern for clinicians. A high index of clinical suspicion towards skin malignancy is essential, and further referral to dermatologists should be the next step since this type of case warrants a more thorough assessment. A skin biopsy should be performed to establish the diagnosis and avoid delaying treatment. Thus, this case report emphasises the importance of considering hypopigmented MF in patients exhibiting longstanding hypopigmented skin lesions without a clear cause to prevent unnecessary treatment and progression to more advanced stages.
